# A Novel Scaffold-Based Hybrid Multicellular Model for Pancreatic Ductal Adenocarcinoma—Toward a Better Mimicry of the *in vivo* Tumor Microenvironment

**DOI:** 10.3389/fbioe.2020.00290

**Published:** 2020-04-24

**Authors:** Priyanka Gupta, Pedro A. Pérez-Mancera, Hemant Kocher, Andrew Nisbet, Giuseppe Schettino, Eirini G. Velliou

**Affiliations:** ^1^Bioprocess and Biochemical Engineering Group (BioProChem), Department of Chemical and Process Engineering, University of Surrey, Guildford, United Kingdom; ^2^Department of Molecular and Clinical Cancer Medicine, University of Liverpool, Liverpool, United Kingdom; ^3^Centre for Tumour Biology and Experimental Cancer Medicine, Barts Cancer Institute, Queen Mary University of London, London, United Kingdom; ^4^Department of Medical Physics and Biomedical Engineering, University College London, London, United Kingdom; ^5^Department of Physics, University of Surrey, Guildford, United Kingdom; ^6^Medical Radiation Science Group, The National Physical Laboratory, Teddington, United Kingdom

**Keywords:** pancreatic cancer, multicellular tumor model, 3D model, endothelial cells, pancreatic stellate cells, scaffold-assisted tumor model, polyurethane scaffold

## Abstract

With a very low survival rate, pancreatic ductal adenocarcinoma (PDAC) is a deadly disease. This has been primarily attributed to (i) its late diagnosis and (ii) its high resistance to current treatment methods. The latter specifically requires the development of robust, realistic *in vitro* models of PDAC, capable of accurately mimicking the *in vivo* tumor niche. Advancements in the field of tissue engineering (TE) have helped the development of such models for PDAC. Herein, we report for the first time a novel hybrid, polyurethane (PU) scaffold-based, long-term, multicellular (tri-culture) model of pancreatic cancer involving cancer cells, endothelial cells, and stellate cells. Recognizing the importance of ECM proteins for optimal growth of different cell types, the model consists of two different zones/compartments: an inner tumor compartment consisting of cancer cells [fibronectin (FN)-coated] and a surrounding stromal compartment consisting of stellate and endothelial cells [collagen I (COL)-coated]. Our developed novel hybrid, tri-culture model supports the proliferation of all different cell types for 35 days (5 weeks), which is the longest reported timeframe *in vitro*. Furthermore, the hybrid model showed extensive COL production by the cells, mimicking desmoplasia, one of PDAC’s hallmark features. Fibril alignment of the stellate cells was observed, which attested to their activated state. All three cell types expressed various cell-specific markers within the scaffolds, throughout the culture period and showed cellular migration between the two zones of the hybrid scaffold. Our novel model has great potential as a low-cost tool for *in vitro* studies of PDAC, as well as for treatment screening.

## Introduction

Pancreatic ductal adenocarcinoma (PDAC) is the fourth leading cause of cancer-related deaths worldwide and accounts for about 7% of all cancer-related deaths (Siegel et al., 2018). The 5-year survival rate is about 9% and has barely improved over the last decades (Cancer.Net, 2019). These dismal figures for PDAC are due to its late-stage diagnosis, early and rapid metastasis, along with a high resistance to currently available treatment options (mainly, chemotherapy and radiotherapy) (Kleeff et al., 2016). The latter is attributed to the complex tumor microenvironment (TME) of PDAC. The PDAC’s TME consists of a cocktail of cellular, biochemical, biomechanical, and structural components, which interact in complex ways and contribute to the disease progression. More specifically, the stellate cells of the TME are known to produce very high amounts of extracellular matrix (ECM) proteins, leading to the so-called desmoplastic or fibrotic reaction. The increase of matrix proteins, e.g., collagen, fibronectin (FN), also results in tumor vessel collapse, along with the formation of aberrant, disorganized vessel networks (Longo et al., 2016). Overall, fibrosis/desmoplasia contributes to the high resistance of PDAC to treatment (Seicean et al., 2015; Chand et al., 2016; Totti et al., 2017; Ansari et al., 2018; Totti et al., 2018).

Traditionally, research on PDAC is conducted in (i) 2D *in vitro* systems (Onishi et al., 2012; Sato et al., 2018; Zhang et al., 2018; Serri et al., 2019) or in (ii) animal models, primarily mice (Awasthi et al., 2011; Dovzhanskiy et al., 2012; Courtin et al., 2013; Shinoda et al., 2018; Zhang et al., 2018; Awasthi et al., 2019). Although 2D systems are cheap, easy to use, and reproducible, they are unable to mimic accurately key *in vivo* characteristics like the TME structure, stiffness, the cellular spatial orientation, the cellular cross-talk, the cell-ECM interactions, or the environmental gradients (Onishi et al., 2012; Adcock et al., 2015; Jaidev et al., 2015; Totti et al., 2017; Chim and Mikos, 2018). Animal models can accurately mimic the *in vivo* conditions and hence are widely used for laboratory research and pre-clinical trials (Pérez–Mancera et al., 2012; Courtin et al., 2013; Bermejo-Rodríguez and Pérez-Mancera, 2015; Erstad et al., 2018; Humpton et al., 2019; Yan et al., 2019). However, such systems are expensive, difficult to use, and are not easily reproducible (Pérez–Mancera et al., 2012; Adcock et al., 2015; Ireland et al., 2016; Yan et al., 2019).

Advancements in the field of tissue engineering (TE) have enabled the development of different types of 3D *in vitro* models that realistically mimic *in vivo* tissue niches, including tumor tissues. Current 3D models of pancreatic tumors include (i) spheroids (from cell lines) or organoids (from primary tissue) (Froeling et al., 2009; Matsuda et al., 2010; Longati et al., 2013; Wen et al., 2013; Boj et al., 2015; Chiellini et al., 2016; Di Maggio et al., 2016; Ware et al., 2016; Brancato et al., 2017), (ii) hydrogels (Ki et al., 2014; Chiellini et al., 2016; Brancato et al., 2017; Okumura et al., 2019), and (iii) polymeric scaffolds based systems (He et al., 2013; Raza et al., 2013; Wang et al., 2013; Ricci et al., 2014; Chand et al., 2016; Totti et al., 2018). Overall, such 3D models have substantial advantages as compared to 2D systems and animal models. These include low cost and higher reproducibility, as compared to animal models and provision of more realistic structure, cell–cell and cell–ECM interactions, and realistic distribution of parameters, such as nutrients and oxygen concentration, as compared to 2D systems (Fernandes et al., 2009; Wang et al., 2016; Totti et al., 2017). For example, Longati et al. (2013) showed increased matrix protein secretion and increased resistance to the chemotherapeutic agent Gemcitabine in 3D spheroids, as compared to 2D systems for PANC-1 pancreatic cancer cell lines. Similarly, an increase in chemo-resistance in 3D spheroids when compared to 2D was also reported by Wen et al. (2013) for PANC-1 and MIA PaCa-2 cell lines. Ki et al. (2014) encapsulated COLO-357 cells within poly(ethylene glycol)-based hydrogels enhanced with collagen I (COL) fibrils to mimic the PDAC’s desmoplasia and observed enhanced cell proliferation and epithelial–mesenchymal transition (EMT) within gels enriched with COL. Long-term (i.e., some weeks), culture of pancreatic cancer cells within polymeric scaffolds and hydrogels has been reported in some studies (Ricci et al., 2014; Chiellini et al., 2016; Totti et al., 2018; Gupta et al., 2019). Chiellini et al. carried out long-term (28 days) culture of BxPC-3 cell lines within micro-structured chitosan (mCS)-based or polyelectrolyte complex (mPEC) hydrogels. It was reported that cells in the hydrogels were able to maintain cancer features, like loss of cell polarity, which were not present in 2D. Furthermore, increase in matrix stiffness enhanced the expression of tumor-specific markers (Chiellini et al., 2016). We have also recently reported long-term (more than 5 weeks) culture of various PDAC cell lines, i.e., PANC-1, AsPC-1, BxPC-3, in polyurethane (PU) polymeric scaffolds wherein cell clustering, cell proliferation, and matrix protein production followed *in vivo*-like trends (Totti et al., 2018). We also reported that the model was able to mimic a clinically relevant response to various treatment protocols (Gupta et al., 2019).

However, all of the above models are monocellular, taking into consideration only pancreatic cancer cells. Therefore, they cannot recapitulate accurately the cellular complexity of the PDAC TME, which contains a plethora of different cell types, e.g., endothelial cells, stellate cells, which are crucial for the disease progression and resistance to treatment (Wehr et al., 2011; Hamada et al., 2012; Karnevi et al., 2016; Bynigeri et al., 2017). It is therefore important to recapitulate, in addition to the structural and biochemical complexity, features of the biological complexity of the PDAC TME. There are very limited multicellular 3D PDAC models, such as spheroids/organoids or hydrogel-based systems (Froeling et al., 2009; Di Maggio et al., 2016; Longo et al., 2016; Priwitaningrum et al., 2016; Ware et al., 2016; Brancato et al., 2017; Kuen et al., 2017; Noel et al., 2017; Shoval et al., 2017; Lazzari et al., 2018). Most multicellular PDAC models consist of two cells types involving cancer cells co-cultured with fibroblasts/stellate cells, endothelial cells, mesenchymal stem cells (MSCs), or immune cells. For example, Froeling et al. used COL and Matrigel to create spheroids of pancreatic cancer cells (Capan-1 and PaCa-3) with activated stellate cells or the normal fibroblastic cell line MRC-5 for 7 days. An increase in the number of invasive cancer cells and a decrease in the expression of cytokeratin (suggesting EMT) was observed in presence of stellate cells and MRC-5 fibroblasts (Froeling et al., 2009). Similarly, Drifka et al. employed a collagen-coated microchannel spheroid-based co-culture of cancer cells (PANC-1) and primary stellate cells. Stellate cells facilitated collagen fiber alignment and helped cancer cell migration through the matrix (Drifka et al., 2016). Kuen et al. showed that the co-culture of cancer cells (PaTu-8902, BxPC-3, HPAC, and MiaCaPa-2) and MRC-5 fibroblasts in a spheroid model induced the production of immunosuppressive cytokines, highlighting the immunosuppressive role of different cell types within the tumor niche (Kuen et al., 2017). Ware et al. (2016) observed an impaired diffusion of Gemcitabine (1000 μM) in PDAC spheroids (PANC-1, AsPC-1, BxPC-3, Capan-1, and MIA PaCa-2) when they contained primary stellate cells as compared to mono-cellular cancer cell spheroids. Similarly, an increased resistance to oxaliplatin treatment in co-culture of patient-derived cancer associated fibroblasts (CAFs) with pancreatic cancer cells (MIA PaCa-2 and AsPC-1) in spheroids was observed by Broekgaarden et al. (2019). There are very few studies reporting co-culture of cancer cells with endothelial cells. Shoval et al. (2017) performed a 72-h co-culture with BxPC-3 PDAC cells and endothelial cells (HUVECs) in a spheroid model, wherein it was shown that the HUVECs mainly grew at the periphery of the spheroids and were unable to form vascular structures within the spheroids.

Among the multicellular PDAC studies, there are very limited studies of PDAC involving the presence of three cell types, and all those studies are in spheroid-type systems for a relatively short time period (24 h to 7 days). For example Beckermann et al. (2008) co-cultured pancreatic cancer cells (Capan-1, MIA-PaCa2, COLO-357, and BxPC-3), endothelial cells (HUVECs), and normal primary fibroblast cells in a spheroid model for 24 h. Di Maggio et al. (2016) developed a spheroid (Matrigel and COL-assisted)-based tri-culture model involving cancer cells (Capan-1, COLO-357, and AsPC-1), HUVECs, and activated pancreatic stellate cells (PS-1) and cultured it for 7 days. A gradual depletion of CD-31 positive HUVECs was observed in the spheroid system over time. Similarly, Lazzari et al. developed a co-culture model, which included cancer cells (PANC-1), HUVECs, and the fibroblast cell line MRC-5 for a period of 7 days. No endothelial cells were observed in the system after 4 days in culture. Furthermore, higher resistance to gemcitabine and doxorubicin was observed in the multicellular spheroids as compared to the monocellular ones (Lazzari et al., 2018).

Overall, spheroid-type multicellular models are valuable and suitable for molecular analysis and for fast drug response studies; however, they have certain limitations. Due to their spatial characteristics, artificially high diffusion gradients in terms of nutrients and oxygen can be formed, resulting in necrotic cores at the center and decreasing cellular proliferation very quickly (within a few days) (Burdett et al., 2010; Nath and Devi, 2016; Totti et al., 2017). Consequently, they are difficult to maintain over a long period of time (weeks or months) without re-suspending the cells to form fresh cellular aggregates. Such re-suspension can disturb the formed TME and cell–cell, cell–ECM interactions. Furthermore, it is difficult to robustly control the spheroid size and shape (Burdett et al., 2010; Nath and Devi, 2016; Totti et al., 2017). Hydrogel-type spheroids have better structure than simple cell-aggregates; however, they have relatively weak mechanical strength, making their long-term maintenance in culture challenging (Hoffman, 2012; Totti et al., 2017).

Polymer scaffold-assisted 3D structures can overcome several of the limitations associated with spheroids and hydrogels. They can provide a more robust mechanical strength and tunability allowing for much longer cultures (up to months), and they can be tuned to have appropriate internal structure, pore size, type, and distribution, enabling the recapitulation of the spatial organization of different cell types in a multicellular system, as well as allowing for proper diffusion of oxygen and other nutrients (O’Brien, 2011; Ricci et al., 2014; Velliou et al., 2015; Totti et al., 2017, 2018; Gupta et al., 2019). To the best of our knowledge, to date, there is no scaffold-assisted, multicellular model for PDAC.

The aim of this work was to address the above challenge *via* the development of a novel, multicellular, hybrid, PU scaffold-based model involving PANC-1 cancer cells, human microvascular endothelial cells (HMECs), and PS-1 pancreatic stellate cells. More specifically, building on our previously developed monocellular PU scaffold (Totti et al., 2018; Gupta et al., 2019), we performed appropriate zonal surface modification of the scaffolds with FN or COL to support growth and proliferation of different cells of the PDAC TME and we monitored proliferation, spatial organization, ECM secretion, and cellular interactions for a total of 5 weeks.

## Materials and Methods

### Polymer Scaffold Preparation and Surface Modification

Polyurethane scaffolds were fabricated via the thermal induced phase separation method, as reported previously (Velliou et al., 2015; Totti et al., 2018). The scaffolds were then cut at appropriate sizes (see sections “Single Scaffold-Based 3D Cell Culture” and “Scaffold-Based Zonal 3D Cell Culture”) and sterilized by exposing them to 70% ethanol (3 h) and UV ray (1 h). As previously reported, the average pore size of the scaffolds was 100–150 μm, the porosity was 85–90%, and the elastic modulus, 20 ± 2 kPa. It should be stated that the stiffness of the scaffolds was similar to that of PDAC *ex vivo* tissue (Chantarojanasiri and Kongkam, 2017; Pozzi et al., 2017; Rice et al., 2017).

Thereafter, as previously described, the generated scaffolds were surface modified (adsorption) with FN or COL for ECM mimicry (Totti et al., 2018; Gupta et al., 2019).

### 2D Cell Culture

The human pancreatic adenocarcinoma cell line PANC-1 (Sigma–Aldrich, Merck, United Kingdom) was expanded in Dulbecco’s modified Eagle’s medium (DMEM) with high glucose (Sigma–Aldrich, Merck, United Kingdom) supplemented with 10% fetal bovine serum (FBS, Fisher Scientific, United Kingdom), 1% penicillin/streptomycin (Fisher Scientific, United Kingdom), and 2 mM L-glutamine (Sigma–Aldrich, Merck, United Kingdom) in a humidified incubator at 37°C with 5% CO_2_.

The HMEC line CRL*-3243* (ATCC, United Kingdom) was expanded in MCDB 131 medium (GIBCO, Thermo Fisher, United Kingdom), supplemented with 10% FBS, 1% penicillin/streptomycin, 2 mM L-glutamine, 10 ng/ml epidermal growth factor (SIGMA–Aldrich, Merck, United Kingdom), and 1 μg/ml hydrocortisone (SIGMA–Aldrich, Merck, United Kingdom) in a humidified incubator at 37°C with 5% CO_2_.

The immortalized human pancreatic stellate cells (PS-1) were expanded in DMEM/F12 medium (GIBCO, Thermo Fisher, United Kingdom) supplemented with 10% FBS, 1% penicillin/streptomycin, and 2 mM L-glutamine in a humidified incubator at 37°C with 5% CO_2_.

All cells were passaged regularly on reaching 80–90% confluency with TrypLE (GIBCO, Thermo Fisher, United Kingdom) until the required cell densities were obtained.

### 3D Cell Culture

#### Single Scaffold-Based 3D Cell Culture

Uncoated, FN- or COL-coated scaffolds were tested to analyze their ability to support PS-1 and HMEC cells in mono-culture, co-culture (PANC-1 + HMEC or PANC-1 + PS-1), and tri-culture (PANC-1 + PS-1 + HMEC).

For mono-culture experiments, 0.5 × 10^6^ cells were seeded in each scaffold (5 × 5 × 5-mm^3^-sized) (re-suspended in a total of 30 μl of cell culture media per scaffold) (Totti et al., 2018; Gupta et al., 2019). For the co-culture and tri-culture experiments, 0.25 × 10^6^ cells per cell type were seeded in each scaffold (5 × 5 × 5-mm^3^-sized), placed in 24 well plates, and cultured for 28 days (4 weeks), as per our previously established protocol (Totti et al., 2018; Gupta et al., 2019).

#### Scaffold-Based Zonal 3D Cell Culture

**FIGURE 1:**
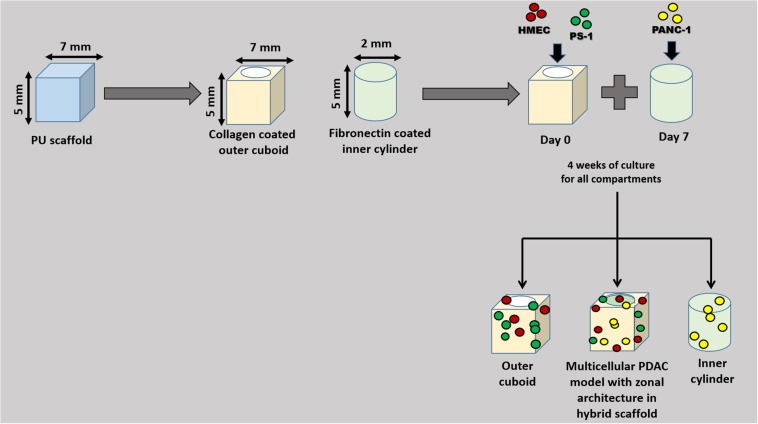
Schematic diagram of the zonal architecture development for the scaffold-assisted multicellular model of PDAC. Polyurethane (PU) scaffolds were appropriately cut to design the zonal architecture. Different cells types were seeded at different time points and at different locations of the scaffold. The tri-culture system was monitored for 28 days (for a total experimental period of 35 days).

**FIGURE 2:**
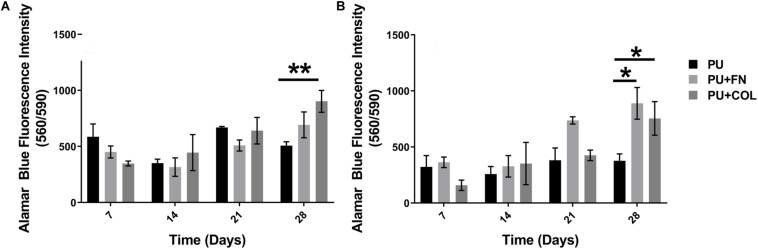
Overall cellular metabolic activity as determined by the Alamar Blue metabolic assay within different PU scaffolds configurations (uncoated-PU, fibronectin-FN-coated, and collagen-COL-coated). **(A)** HMEC. **(B)** PS-1. *p ≤ 0.05, **p ≤ 0.01.

The single scaffold-based analysis for mono-, co-, and tri-cultures showed that different cell types prefer different ECM presence on the scaffold surface (see section “Results”). Therefore, to recapitulate that, a zonal scaffold architecture was designed. More specifically, as shown in Figure 1, two separate zones (a hollow cuboid with dimensions of approximately 7 × 7 × 5 mm^3^ and a solid inner cylinder of diameter of approximately 2 mm and height of 5 mm) were created/cut from the PU scaffold (prepared as described in section “Polymer Scaffold Preparation and Surface Modification”) using a biopsy punch. The outer cuboid was coated with COL, while the inner cylinder was coated with FN through passive absorption, as described in Section “Polymer Scaffold Preparation and Surface Modification.” HMEC and PS-1 stellate cells were seeded into the hollow cuboid in different ratios. As previously described, immediately after seeding, the scaffolds were placed in the incubator and cultured per our established protocol (Totti et al., 2018; Gupta et al., 2019) (section “Single Scaffold-Based 3D Cell Culture”). Based on our monocellular studies (Figure 2; Totti et al., 2018), we observed that PANC-1 cancer cells expanded at a faster rate in comparison to PS-1 and HMEC cells. Hence, to avoid the cancer cells’ over-growing, as compared to the endothelial and stellate cells, we cultured the supporting cells (PS-1 and HMEC) for 7 days. On day 7, PANC-1 cells were seeded into the solid inner cylinder in a similar manner and then plugged inside the hollow cuboid to assemble to complete hybrid zonal model. The final ratios tested for PANC-1: HMEC: PS-1 were 1:1:1, 1:2:2, and 1:2:9, based on both ratios reported in literature for spheroid systems and on our initial trials (see single-scaffold-based experiments in sections “Single Scaffold-Based 3D Cell Culture” and “Scaffold-Based Zonal 3D Cell Culture”) (Froeling et al., 2009; Di Maggio et al., 2016; Lazzari et al., 2018). Thereafter, the tri-culture was monitored for an additional 28 days (4 weeks). Separate inner and outer scaffold compartments were also cultured for the same duration of the experiment as controls for the individual zones.

### Alamar Blue Viability Assay

The Alamar Blue assay was carried out every week per the manufacturer’s instructions, to assess the cellular metabolic activity of the 3D cultures. Briefly, 10% Alamar Blue (Thermo Scientific, United Kingdom) solution was prepared in complete cell culture medium and added to the scaffolds followed by 2–3 h incubation at 37°C. At the end of the incubation period, change in Alamar Blue fluorescence was measured using BioTek, Plate reader (BioTek, United Kingdom) at 530 nm excitation and 590 nm emission.

### Immunofluorescence Assay

*In situ* immunofluorescence (IF) staining of the scaffolds took place for the spatial determination of (i) the different cell types, CD-31 (HMEC), αSMA (PS-1), and pan-Cytokeratin (PANC-1), (ii) the cell proliferation (Ki-67), and (iii) the ECM production (COL). More specifically, scaffolds were snap frozen at specific time points in liquid nitrogen for 15 min and then preserved at -80°C until sectioning, as previously described (Allenby et al., 2017, 2019; Tahlawi et al., 2019). Prior to IF staining, scaffolds were sectioned and fixed for 4 h in 4% w/v paraformaldehyde (Sigma–Aldrich, Merck, United Kingdom). For intracellular proteins, scaffold sections were permeabilized for 2 h with 0.1% Triton-X solution (Sigma–Aldrich, Merck, United Kingdom), followed by 3 h blocking using 10% donkey serum solution. For membrane associated proteins, blocking was carried out without permeabilization. The primary antibody staining was carried out overnight, followed by overnight secondary antibody and DAPI co-staining. Each step employed a solvent containing 1% w/v bovine serum albumin (Sigma–Aldrich, Merck, United Kingdom) and 0.5% v/v Tween-20 (Promega, United Kingdom). For multi-panel staining involving both cell membrane and intracellular proteins, blocking, primary, secondary and DAPI staining solutions were made using 1% BSA, 0.5% Tween-20, and 0.1% Saponin (SIGMA–Aldrich, Merck, United Kingdom) solution to facilitate gentle permeabilization without the use of Triton-X.

### Confocal Laser Scanning Microscopy (CLSM) Imaging

Immunofluorescent samples were imaged with a Nikon Ti-Eclipse inverted confocal microscope (Nikon Instruments, Europe) and processed with the NIS-Elements software using the following lasers and filters: (i) 405 (for DAPI), (ii) 488 (for Alexa Fluor 488, Dylight 488), (iii) 561 (for Alexa Fluor 555, Dylight 550), and (iv) 643 nm (for Alexa Fluor 647, Dylight 650) for two sequential scans. Confocal images were captured using a 10x dry objective, with a 512 × 512-pixel resolution and 5–10 μm Z-stack distance, as previously described (Totti et al., 2018; Gupta et al., 2019). Multiple scaffolds as well as multiple areas and sections per scaffold were imaged to ensure reproducibility. Representative images are presented in this manuscript.

### Statistical Analysis

Statistical analysis was performed for at least three independent experiments (*n* ≥ 3) with at least three replicates per time point. Analysis of variance (ANOVA), followed by the Tukey’s multiple comparison test, using the Graph Pad Prism^®^ software (version 8.00 for Windows) to determine data statistical significance (*p* < 0.05). The error bars in the graphs represent standard error of mean.

## Results

### Long-Term Mono-Culture of Stellate Cells and Endothelial Cells on PU Scaffolds

We have previously reported that PANC-1 pancreatic cancer cells are able to grow on PU scaffolds for over 28 days (4 weeks), forming dense cell clusters and secreting substantial amounts of COL in FN coated scaffolds (Totti et al., 2018). Similarly, in this work, mono-cultures of HMEC endothelial cells and PS-1 stellate cells were established on PU scaffolds both uncoated and coated with either FN or COL for 28 days (section “Single Scaffold-Based 3D Cell Culture”). Cell growth and viability were assessed weekly using the Alamar Blue viability assay (section “Alamar Blue Viability Assay”). As observed in Figure 2, both HMEC and PS-1 cells were able to attach and grow on the PU scaffolds for 28 days. At the end of 28 days, HMEC showed significantly higher cell viability on COL-coated scaffolds in comparison to uncoated ones. PS-1 stellate cells showed a significantly higher preference for coated scaffolds (FN or COL) over uncoated ones in terms of cellular metabolic activity as measured by the Alamar Blue assay (Figure 2).

### Single PU Scaffold-Based Co-Culture and Tri-Culture of Stellate (PS-1), Endothelial (HMEC), and Cancer Cells (PANC-1)

**FIGURE 3:**
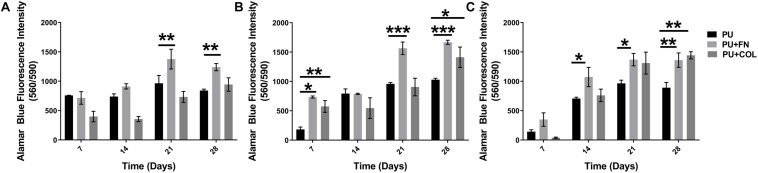
Overall cellular metabolic activity as determined by the Alamar Blue metabolic assay within different PU scaffolds configurations (uncoated, fibronectin-FN-coated, and collagen-COL-coated). **(A)** PANC-1 + HMEC. **(B)** PANC-1 + PS-1. **(C)** PANC-1 + HMEC + PS-1. *p ≤ 0.05, **p ≤ 0.01, ***p ≤ 0.001.

As observed in the mono-culture experimental systems (section “Long-Term Monoculture of Stellate Cells and Endothelial Cells on PU Scaffolds”), both the PS-1 and HMEC cells were able to grow on PU scaffolds for 28 days and showed a preference for ECM protein coated scaffolds in comparison to those uncoated (Figure 2). Also, in our previously published work (Totti et al., 2018), we have reported that PANC-1 cells were able to grow on PU scaffolds (both coated and uncoated) for 28 days (4 weeks), with higher proliferation being observed in FN-coated scaffolds. Therefore, based on the results of the mono-cultures, we established co-culture and tri-culture systems using PU scaffolds, either uncoated or coated (FN or COL). Protein coatings enable the determination of the effects of different ECM proteins on such complex multicellular 3D models. As described in section “Single Scaffold-Based 3D Cell Culture,” different combinations of the three cell types (PANC-1 + HMEC, PANC-1 + PS-1, and PANC-1 + HMEC + PS-1) were added to the scaffolds and cultured for 28 days. The overall cellular metabolic activity as an indication of the overall cell viability was monitored at regular intervals *via* the Alamar Blue Viability Assay (Figure 3).

**FIGURE 4:**
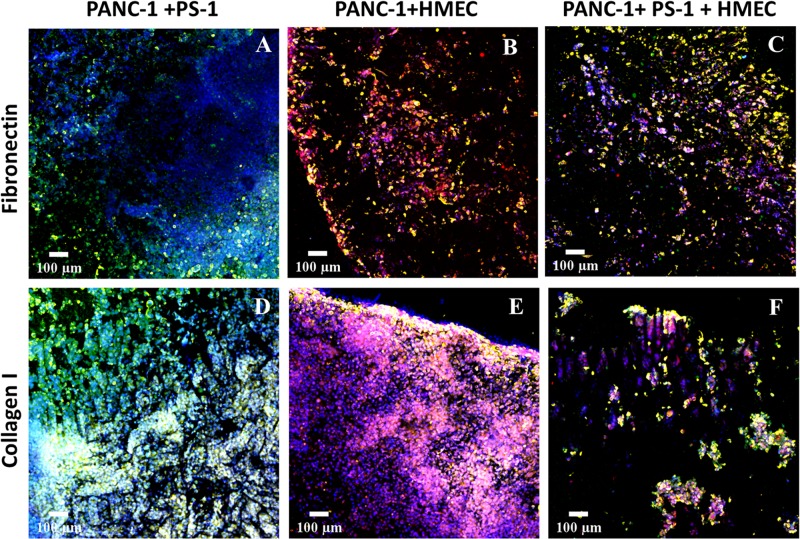
Representative immunofluorescence CLSM images of sections of the 3D scaffolds after 28 days (4 weeks) of culturing multiple cell types in fibronectin-coated **(A–C)** and collagen I-coated **(D–F)** coated scaffolds: PANC-1 PDAC cell lines are in yellow (pan-Cytokeratin staining), PS-1 stellate cells are in green (αSMA staining), and HMEC endothelial cells are in red/pink (CD-31 staining). All cells were stained with DAPI, as well (blue). Scale bar = 100 μm.

As can be seen in Figure 3, the co-cultures as well as the tri-culture involving PANC-1, PS-1, and HMEC cells were all viable throughout the duration of our experiment (28 days). Significantly higher number of viable cells were observed on PU scaffolds coated with FN or COL in comparison to the uncoated scaffolds, similar to the HMEC and PS-1 mono-cultures (Figure 2; section “Long-Term Monoculture of Stellate Cells and Endothelial Cells on PU Scaffolds”), as well as in comparison to our previously published work for cancer cells (PANC-1) mono-culture (Totti et al., 2018). At the end of 28 days, sectioning and *in situ* fluorescence imaging of different cell-specific markers was conducted (i) to monitor the growth of all different cell types and (ii) to enable the assessment of the cell spatial distribution within the scaffolds (Figure 4). More specifically, HMEC cells were identified by CD-31 marker, stellate cells were identified by αSMA, and PANC-1 cells were stained for pan-Cytokeratin (section “Immunofluorescence Assay”). It is worth pointing out that most cancer cell lines are a heterogeneous mixture of cells at different stages of differentiation, hence not all cancer cells express the same proteins/markers (in this case, pan-Cytokeratin). Therefore, for our confocal laser scanning microscopy (CLSM) imaging in co and tri-culture systems, cells that only showed DAPI (nucleus) staining and no cell specific markers are assumed to be PANC-1 cancer cells.

As seen in Figure 4, all three cell types—i.e., cancer, endothelial, and stellate cells—were present within the PU scaffolds at the end of the 28-day culture period for both ECM coatings. The growth rate though of different cell types varied depending on the coating. For example, although FN-coated scaffolds promoted the growth of all cell types (both in co- and tri-culture systems, Figures 4A–C), for the co-culture of PANC-1 cancer cells and PS-1 stellate cells, the growth of PANC-1 was higher as compared to PS-1 cells (Figure 4A). More specifically, the PS-1 stellate cells were mainly found toward the periphery of the model, while PANC-1 cells were distributed throughout the whole scaffold. In contrast, COL-coating helped in a more homogenous growth and distribution of PS-1 stellate cells in a PANC-1 and PS-1 co-culture system (Figure 4D). The co-culture of PANC-1 cancer cells and HMEC endothelial cells also showed a similar trend. FN-coated scaffolds promoted the growth of PANC-1 over HMEC cells (Figure 4B), although in contrast to PS-1 (Figure 4A), HMEC cells were more evenly distributed within the FN-coated scaffolds (Figure 4B). COL-coating showed a significant increase in the number of endothelial cells within the scaffold resulting in dense cellular clusters (Figure 4E), clearly highlighting HMEC cells’ preference for COL matrix protein. Nonetheless, both FN- and COL-coating were able to support a tri-culture tumor model within the PU scaffolds (Figures 4C,F). Similar to the co-cultures (Figures 4A,B), FN-coated PU scaffolds favored PANC-1 cancer cells over the HMEC and PS-1 cells (Figure 4C). The growth of the stellate cells was particularly suppressed within this system. In contrast, the COL-coated scaffolds promoted the growth of HMEC and PS-1 cells, resulting in a more homogenous distribution of all three cells types within the tumor model (Figure 4F).

### PU Scaffold-Based Hybrid Zonal Multicellular Model of PDAC With Tri-Culture of Stellate (PS-1), Endothelial (HMEC), and Cancer Cells (PANC-1)

Overall, our observations on the co-culture and tri-culture systems above [section “Single PU Scaffold-Based Co-Culture and Tri-Culture of Stellate (PS-1), Endothelial (HMEC), and Cancer Cells (PANC-1)”], highlighted that the cellular interactions and cellular growth rates of different cell types in a mixed culture are affected by the ECM protein coating of the PU scaffolds. Specifically, for our PDAC model, PANC-1 cancer cells prefer FN coating, while the HMEC endothelial cells prefer COL. PS-1 stellate cells prefer coated scaffolds, both FN and COL, over uncoated ones. Thus, we further designed a hybrid zonal PU scaffold-based model with different ECM-coatings (Figure 1). More specifically, as described in Section “Scaffold-Based Zonal 3D Cell Culture,” PS-1 and HMEC cells were cultured in a COL-coated external scaffold (stromal compartment), while PANC-1 was grown in an FN-coated inner scaffold (tumor compartment). This configuration enabled (i) tailoring of the ECM to the cell needs and (ii) a better zonal recapitulation of the cell distribution in the PDAC TME. The zonal model was monitored and analyzed at a compartmental level and as a whole (both compartments). More specifically, the following compartments were monitored: (i) FN-coated inner cylinder compartment containing PANC-1 cancer cells, (ii) COL-coated outer cuboid compartment containing HMEC and PS-1 cells, and (iii) the complete hybrid model containing both the inner and the outer compartment (see also section “Scaffold-Based Zonal 3D Cell Culture” and Figure 1).

#### Fibronectin-Coated PU Inner Cylinder Compartment of the Hybrid Scaffold (Containing PANC-1 Cells)

A mono-culture of PANC-1 cancer cells in the FN-coated inner scaffold compartment was monitored for 28 days, since for our hybrid model PANC-1 cancer cells were added 7 days after the development of the outer cuboid (see also Figure 1 and section “Scaffold-Based Zonal 3D Cell Culture”). Cell proliferation (Ki-67), secretion of ECM (i.e., human specific COL), and expression of cell specific markers (pan-Cytokeratin and CD-24) were assessed at regular intervals via immunostaining and CLSM imaging [see also sections “Immunofluorescence Assay” and “Confocal Laser Scanning Microscopy (CLSM) Imaging”].

**FIGURE 5:**
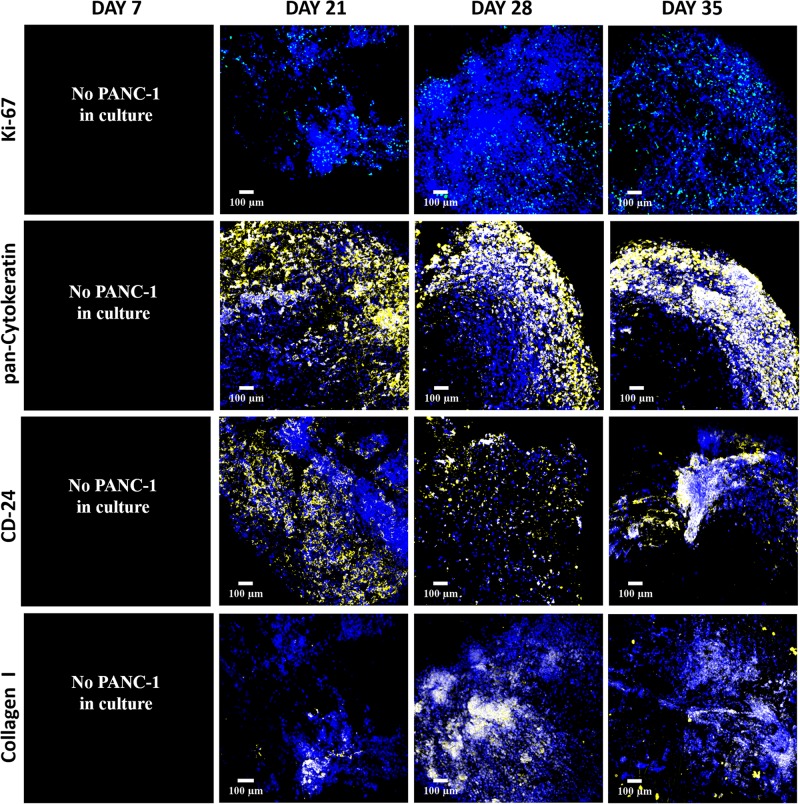
Representative immunofluorescence CLSM images of fibronectin-coated PU inner cylinder of the hybrid scaffold with PANC-1 cells over 28 days of culture. **Top panel:** Ki-67 positive (green) proliferative cells. **Second panel:** Pan-Cytokeratin positive (yellow) PANC-1 cells. **Third panel:** CD-24 positive (yellow) PANC-1 cells. **Bottom panel:** Collagen I secretion (yellow) by the PANC-1 cells. Cell nuclei in all images were stained with DAPI (blue). Scale bar = 100 μm.

As observed in Figure 5, Ki-67 positive proliferative cancer cells were observed throughout the entire culture period (top panel). Multiple PANC-1 cells within the scaffolds expressed both pan-Cytokeratin and CD-24 cellular markers (Figure 5, middle panels), highlighting the heterogeneous nature of the cancer cell population for the PANC-1 cell line (Schüssler et al., 1992; Aghamaliyev et al., 2015; Pei et al., 2016; Haeberle and Esposito, 2019). *In vivo*, COL is overexpressed by pancreatic tumor cells (Imamura et al., 1995) and hence is considered to be an important parameter for the development of a robust *in vitro* model of PDAC. As observed, PANC-1 cells were able to secrete COL within the FN-coated inner compartment of the hybrid scaffold throughout the culture period, the amount increasing with time (Figure 5, bottom panel). Overall, these results highlight that in our FN-coated inner scaffold PANC-1 cancer cells (tumor zone) remain viable, are proliferative and secrete COL throughout the culture period of 28 days.

#### Collagen I-Coated PU Outer Compartment of the Hybrid Scaffold (Containing HMEC Endothelial and PS-1 Stellate Cells)

**FIGURE 6:**
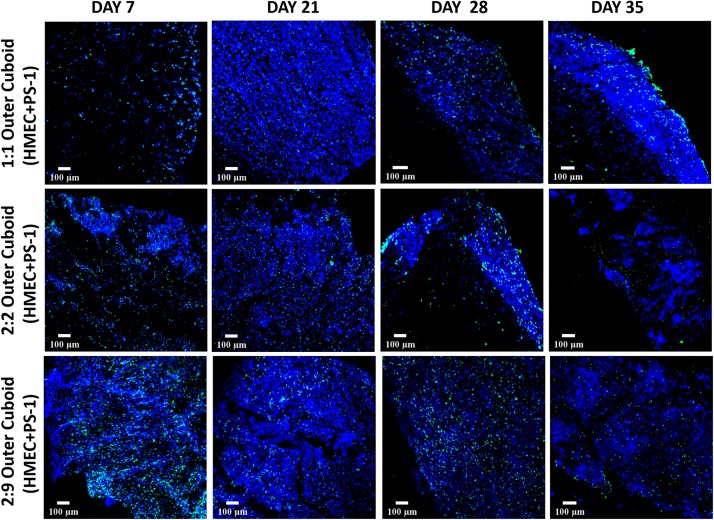
Representative immunofluorescence CLSM images of collagen I-coated PU outer cuboid compartment of the hybrid scaffold with Ki-67 positive (green) proliferative PS-1 and HMEC cells over 35 days of culture. **Top panel:** PS-1:HMEC = 1:1 (PS-1 = 0.25 × 10^6^ cells, HMEC = 0.25 × 10^6^ cells), **Middle panel:** PS-1:HMEC = 2:2 (PS-1 = 0.5 × 10^6^ cells, HMEC = 0.5 × 10^6^ cells), **Bottom panel:** PS1:HMEC = 9:2 (PS-1 = 2.25 × 10^6^ cells, HMEC = 0.5 × 10^6^ cells). Nuclei for all images were stained with DAPI (blue). Scale bar = 100 μm.

Similar to independently studying the inner cylinder of the hybrid scaffold [section “Fibronectin-Coated PU Inner Cylinder Compartment of the Hybrid Scaffold (Containing PANC-1 Cells),” Figure 5] the outer cuboid scaffold consisting of PS-1 and HMEC cells (i.e., recapitulating the stromal compartment of the TME) was independently studied for 35 days (see also Figure 1 and section “Scaffold-Based Zonal 3D Cell Culture”). More specifically, three different ratios of PS-1 and HMEC cells were assessed to study the effect of seeding densities on the evolution of different cells (see also section “Scaffold-Based Zonal 3D Cell Culture”). As previously described [section “Fibronectin-Coated PU Inner Cylinder Compartment of the Hybrid Scaffold (Containing PANC-1 Cells)”], the cellular morphology, cell proliferation, ECM secretion, and cell-specific marker expressions were assessed at regular intervals. As shown in Figure 6, Ki-67 positive proliferative cells were present within the outer scaffold throughout the entire culture period (35 days). Furthermore, at the beginning of the culturing period (day 7), a clear distinction between the different ratios is observed in terms of cell number, i.e., higher seeding density of the HMEC and PS-1 cells showed more proliferating cells. However, by day 21, all three cell ratios under study show a high cell number and a uniform cellular distribution within the scaffolds. However, the number of proliferative cells decreased toward the end of the experimental time (day 35) for all three seeding ratios assessed (Figure 6).

**FIGURE 7:**
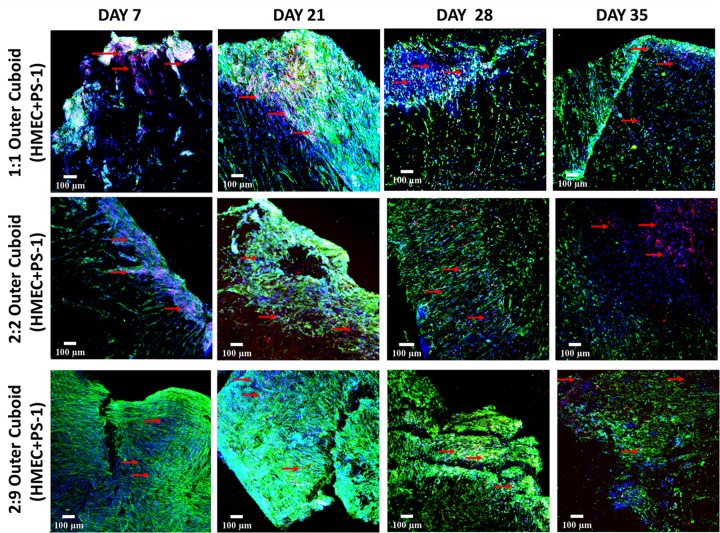
Representative immunofluorescence CLSM images of collagen I-coated PU outer cuboid scaffold compartment of the hybrid scaffold with HMEC (CD-31, red) and PS-1 (αSMA, green) cell distribution over 35 days of culture. **Top panel:** PS-1:HMEC = 1:1 (PS-1 = 0.25 × 10^6^ cells, HMEC = 0.25 × 10^6^ cells), **Middle panel:** PS-1:HMEC = 2:2 (PS-1 = 0.5 × 10^6^ cells, HMEC = 0.5 × 10^6^ cells), **Bottom panel:** PS-1:HMEC = 9:2 (PS-1 = 2.25 × 10^6^ cells, HMEC = 0.5 × 10^6^ cells). Nuclei for all images were stained with DAPI (blue). Endothelial cells are pointed with red arrow. Scale bar = 100 μm.

Cell-specific immunostaining for phenotypic markers was carried out to identify the density and spatial distribution of PS-1 (αSMA) and HMEC (CD-31) cells within the outer cuboid scaffold. Figure 7 shows representative images immunostaining for cell specific markers.

As can be seen in Figure 7, on day 7, the experimental systems with equal number of PS-1 and HMEC cells (Figure 7, top and middle panels) showed relatively similar distribution of the two cell types, while the presence of excess PS-1 in the third experimental system (Figure 7, bottom panel) resulted in the stellate cells growing significantly and suppressing the growth of the HMEC endothelial cells. On day 21 (week 3), all three conditions showed a high number of PS-1 stellate cells. HMEC endothelial cells were mainly visible in conditions with equal ratio of PS-1 and HMEC, although their cell number was generally lower than the PS-1 cells. For the 2:9 (PS-1:HMEC) ratio, similar to day 7, CD-31 positive HMEC cells were not very visible within the co-culture (Supplementary Figure S1). The αSMA-staining also showed fiber-like structure and an aligned nature of the activated stellate cells within the scaffolds, especially for the experiment with abundance of PS-1, i.e., 2:9 ratio. Toward the end of the culture period, at days 28 and 35 (weeks 4 and 5), although cells were present within the scaffolds, their numbers decreased. Furthermore, the morphology of the cells (particularly the stellate cells) changed and loss of cell-specific markers (CD-31 and αSMA) was observed (Figure 7).

Activated pancreatic stellate cells are known to secrete extensive ECM proteins (primarily COL), resulting as previously described, in desmoplasia/fibrosis (Apte et al., 2004; Armstrong et al., 2004; McCarroll et al., 2014). Hence, immunostaining for human-specific COL was carried out for the outer cuboid scaffold.

**FIGURE 8:**
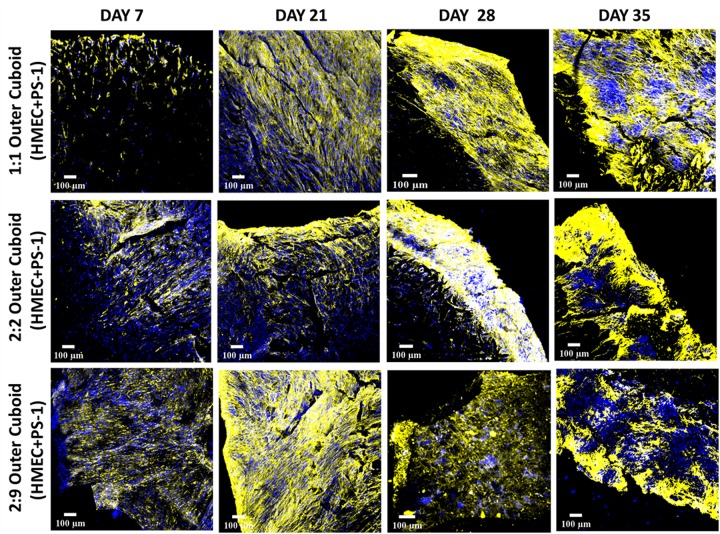
Representative immunofluorescence CLSM images of collagen I (rat tail)-coated PU outer cuboid compartment of the hybrid scaffold for human specific collagen I secretion (yellow) over 35 days of culture. **Top panel:** PS-1:HMEC = 1:1 (PS-1 = 0.25 × 10^6^ cells, HMEC = 0.25 × 10^6^ cells), **Middle panel:** PS-1:HMEC = 2:2 (PS-1 = 0.5 × 10^6^ cells, HMEC = 0.5 × 10^6^ cells), **Bottom panel:** PS-1:HMEC = 9:2 (PS-1 = 2.25 × 10^6^ cells, HMEC = 0.5 × 10^6^ cells). Nuclei for all images were stained with DAPI (blue). Scale bar = 100 μm.

Excessive COL secretion was observed within our outer cuboid scaffold (Figure 8). On day 7, the amount of COL was directly proportional to the number of stellate cells, i.e., higher number of PS-1 resulted in higher amount of COL secretion. For all three conditions, COL secretion increased with time in the early days of the culture (up to day 28). Similar to the cell-specific marker expressions, toward the end of the culture period, a decrease in COL amount was observed (day 35).

#### Complete, Hybrid, Zonal, Multi-Compartmental, Multicellular Model of PDAC Containing Cancer Cells (PANC-1), Endothelial Cells (HMEC), and Stellate Cells (PS-1)

As reported in Sections “Fibronectin-Coated PU Inner Cylinder Compartment of the Hybrid Scaffold (Containing PANC-1 Cells)” and “Collagen I-Coated PU Outer Compartment of the Hybrid Scaffold (Containing HMEC Endothelial and PS-1 Stellate Cells),” both the inner and outer scaffolds of our hybrid model were individually viable for the entire duration of the experiment, i.e., 28 days for the inner scaffold and 35 days for outer scaffold. All three cell types remained in a proliferative state (Figures 5, 6), expressed cell-specific markers (Figures 5, 7), and produced their own COL matrix protein (Figures 5, 8). Thereafter, a multicellular PDAC *in vitro* model was developed by assembling the inner and outer compartments (section “Scaffold-Based Zonal 3D Cell Culture”) to obtain a hybrid, zonal, tri-culture PDAC model containing PANC-1 cancer cells, HMEC endothelial cells, and PS-1 stellate cells (for more details, see section “Scaffold-Based Zonal 3D Cell Culture” and Figure 1). As per the experiments of the separate scaffold compartments [sections “Fibronectin-Coated PU Inner Cylinder Compartment of the Hybrid Scaffold (Containing PANC-1 Cells)” and “Collagen I-Coated PU Outer Compartment of the Hybrid Scaffold (Containing HMEC Endothelial and PS-1 Stellate Cells)”] the cell proliferation, cell-specific marker expression (pan-Cytokeratin, CD-31, and αSMA), and COL secretion in the hybrid scaffold were monitored regularly.

**FIGURE 9:**
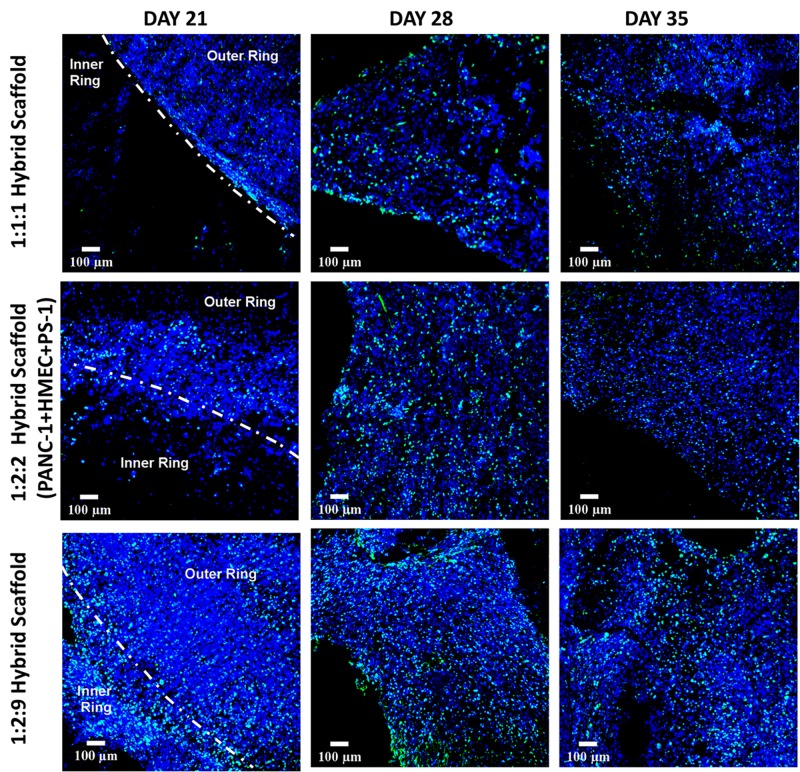
Representative immunofluorescence CLSM images for Ki-67 positive (green) proliferative cells within the complete multicellular hybrid scaffold, containing both the collagen I-coated outer cuboid and the fibronectin-coated inner cylinder, over 35 days of culture. **Top panel:** PANC-1:HMEC:PS-1 = 1:1:1 (PANC-1 = 0.25 × 10^6^ cells, PS-1 = 0.25 × 10^6^ cells, HMEC = 0.25 × 10^6^ cells), **Middle panel:** PANC-1:HMEC:PS-1 = 1:2:2 (PANC-1 = 0.25 × 10^6^ cells, PS-1 = 0.5 × 10^6^ cells, HMEC = 0.5 × 10^6^ cells), **Bottom panel:** PANC-1:HMEC:PS-1 = 1:2:9 (PANC-1 = 0.25 × 10^6^ cells, PS-1 = 2.25 × 10^6^ cells, HMEC = 0.5 × 10^6^ cells). Nuclei for all images were stained with DAPI (blue). Scale bar = 100 μm.

As can be seen in Figure 9, Ki-67 positive proliferative cells were visible in the hybrid scaffold throughout the entire experimental time period, both in the inner and outer scaffold for all seeding ratios (Figure 9). On day 21, outer and inner rings were separately visible (Figure 9, first vertical panel). However, for later time points (i.e., days 28 and 35), the two sections of the scaffolds could not be easily distinguished, especially for the conditions with higher cell numbers, indicating the homogeneous merging of the two compartments (Figure 9).

**FIGURE 10:**
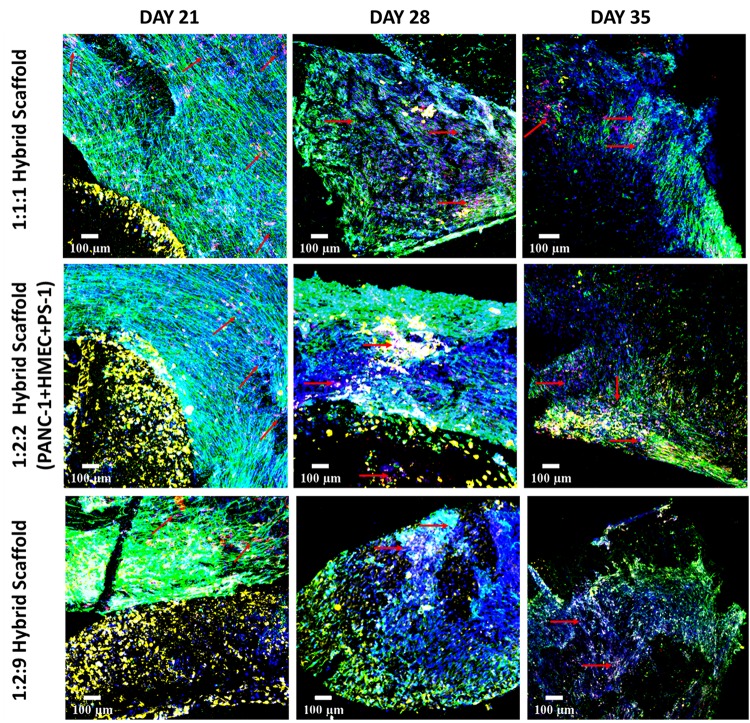
Representative immunofluorescence CLSM images showing the cellular distribution of PANC-1 (pan-Cytokeratin, yellow), HMEC (CD-31, red), and PS-1 (αSMA, green) within the complete hybrid scaffold containing both the collagen I-coated outer cuboid and the fibronectin-coated inner cylinder, over 35 days of culture. **Top panel:** PANC-1:HMEC:PS-1 = 1:1:1 (PANC-1 = 0.25 × 10^6^ cells, PS-1 = 0.25 × 10^6^ cells, HMEC = 0.25 × 10^6^ cells), **Middle panel:** PANC-1:HMEC:PS- = 1:2:2 (PANC-1 = 0.25 × 10^6^ cells, PS-1 = 0.5 × 10^6^ cells, HMEC = 0.5 × 10^6^ cells), **Bottom panel:** PANC-1:HMEC:PS-1 = 1:2:9 (PANC-1 = 0.25 × 10^6^ cells, PS-1 = 2.25 × 10^6^ cells, HMEC = 0.5 × 10^6^ cells). Nuclei for all images were stained with DAPI (blue). Endothelial cells are pointed with red arrow. Scale bar = 100 μm.

As can be seen in Figure 10, for the cell specific phenotypic markers expression, at day 21 (2 weeks post-assembling the hybrid model), all three cell types expressed their specific markers. More specifically, pan-Cytokeratin positive PANC-1 cells were visible within the FN-coated inner cylinder compartment, while an abundance of αSMA positive PS-1 stellate cells was observed in the collagen-coated outer cuboid scaffold compartment. Parallel alignment of the stellate cells was visible for all three seeding densities. CD-31 positive HMEC endothelial cells were present within the dense stellate-cell-rich compartment for all three cell ratios under study (indicated with red arrows in Figure 10), but they were more visible in the experiments where PS-1 and HMEC were in equal numbers (1:1:1 and 1:2:2 hybrid scaffolds). Similar to the experiments of the independent outer cuboid scaffold (Figure 7), changes in cellular morphology and loss of cellular markers were observed on day 28 and were further enhanced at the end of the culture period (day 35).

**FIGURE 11:**
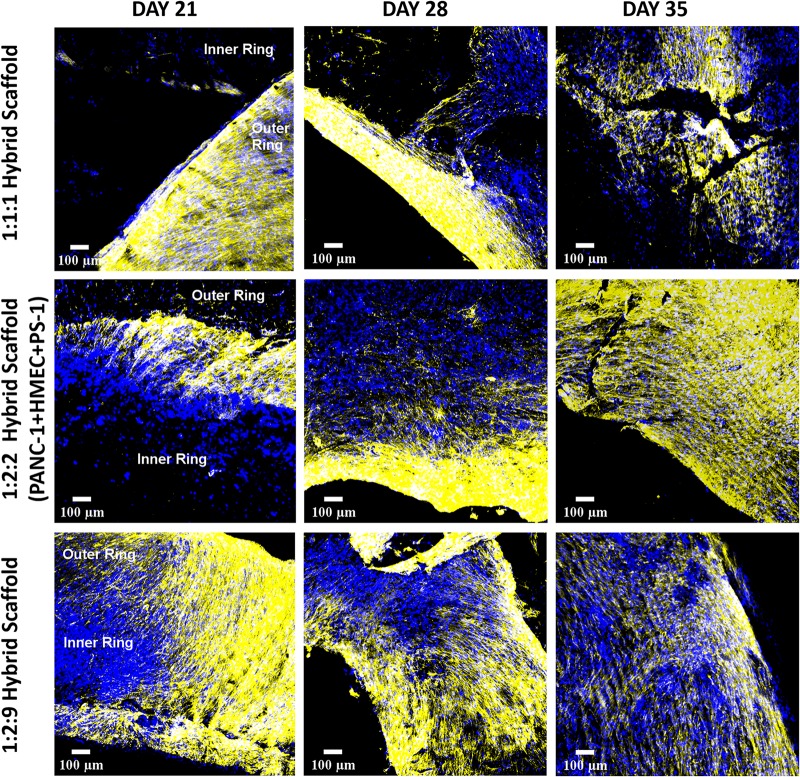
Representative immunofluorescence CLSM images showing collagen I (human) ECM protein secretion within the complete multicellular hybrid scaffold containing both the collagen I (rat tail)-coated outer cuboid and the fibronectin-coated inner cylinder, over 35 days of culture. **Top panel:** PANC-1:HMEC:PS-1 = 1:1:1 (PANC-1 = 0.25 × 10^6^ cells, PS-1 = 0.25 × 10^6^ cells, HMEC = 0.25 × 10^6^ cells), **Middle panel:** PANC-1:HMEC:PS-1 = 1:2:2 (PANC-1 = 0.25 × 10^6^ cells, PS-1 = 0.5 × 10^6^ cells, HMEC = 0.5 × 10^6^ cells), **Bottom panel:** PANC-1:HMEC:PS-1 = 1:2:9 (PANC-1 = 0.25 × 10^6^ cells, PS-1 = 2.25 × 10^6^ cells, HMEC = 0.5 × 10^6^ cells). Nuclei for all images were stained with DAPI (blue). Scale bar = 100 μm.

As mentioned earlier, the fibrotic reaction and the presence of excessive ECM protein (desmoplasia) are hallmarks for PDAC. Hence, COL secretion by the different cells was assessed within our zonal multicellular model (Figure 11). Similar to the separate experiments for the inner and outer scaffold compartments (Figures 5, 8), at the beginning of the culture (day 21), cancer cells in the inner scaffold compartment showed very little COL secretion, while the stellate cells in the outer scaffold compartment showed extensive COL protein production (Figure 11, left panel). As time progressed, more COL secretion was observed by both the PS-1 cells and the PANC-1 cells. At the end of the 35 days (Figure 11, right panel), a slight decrease in COL in the model was observed, in alignment with the loss of cellular marker expressions and the morphology changes (Figure 10).

**FIGURE 12:**
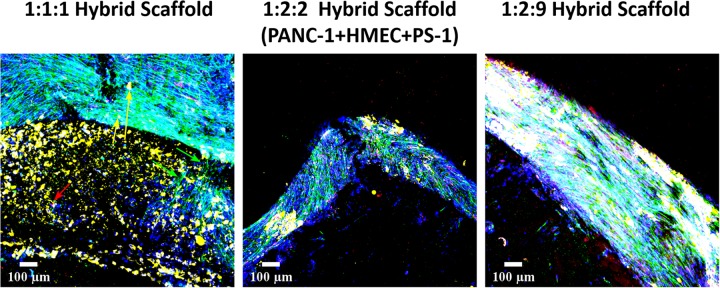
Representative immunofluorescence CLSM image of the hybrid scaffold demonstrating cellular migration of PANC-1 (pan-Cytokeratin, yellow), HMEC (CD-31, red), and PS-1 (αSMA, green) at day 21 (2 weeks post-assembly of hybrid scaffold) between the inner and the outer scaffold compartments. **Left panel:** PANC-1:HMEC:PS-1 = 1:1:1 (PANC-1 = 0.25 × 10^6^ cells, PS-1 = 0.25 × 10^6^ cells, HMEC = 0.25 × 10^6^ cells), **Middle panel:** PANC-1:HMEC:PS-1 = 1:2:2 (PANC-1 = 0.25 × 10^6^ cells, PS-1 = 0.5 × 10^6^ cells, HMEC = 0.5 × 10^6^ cells), **Right panel:** PANC-1:HMEC:PS-1 = 1:2:9 (PANC-1 = 0.25 × 10^6^ cells, PS-1 = 2.25 × 10^6^ cells, HMEC = 0.5 × 10^6^ cells). Nuclei for all images were stained with DAPI (blue). Migration shown by cell-specific arrow: PANC-1 = yellow, PS-1 = green, HMEC = red. Scale bar = 100 μm.

Cellular migration and cellular interactions between the tumor and the stromal cells within a cancer niche are important aspects for cancer metastasis (Keleg et al., 2003; Xu et al., 2010). As our hybrid multicellular model consists of two different scaffold zones/compartments, the ability of the cells, especially the PANC-1 cancer cells to migrate from one compartment to the other is an important requirement for the physiological relevance of the model. As observed in Figure 12, at day 21 (2 weeks post-assembly of the hybrid scaffold), cellular migration was observed for all three cell ratios under study. More specifically, for the 1:1:1 hybrid model (Figure 12, left panel), PANC-1 cells (yellow arrows in Figure 12) migrated from the inner to the outer scaffold compartment containing stellate and endothelial cells while the PS-1 stellate cells (green arrows in Figure 12) bridge the two zones. HMEC (red arrow) migration was also observed. For the 1:2:2 (Figure 12, middle panel) and 1:2:9 (Figure 12, right panel) cell ratios in the hybrid scaffolds, all three cell types (yellow, green, and red) are observed together primarily at the junction of the two scaffold compartments.

Overall, our results show the successful development of a novel hybrid, zonal, multicellular scaffold-based PDAC *in vitro* model containing pancreatic cancer, stellate, and endothelial cells. The model was successfully maintained in culture for a total of 35 days (5 weeks), although cellular/culture aging was observed after 28 days (4 weeks).

## Discussion

Overall, in this work, we developed, characterized, and maintained long-term (35-day) novel PU scaffold-based, multicellular, *in vitro* models of pancreatic cancer consisting of pancreatic cancer (PANC-1), stellate (PS-1), and endothelial (HMEC) cells.

### Single Homogeneous Scaffold-Based Multicellular Model of PDAC

Our novel PU scaffold-based *in vitro* PDAC model was able to maintain cell viability and expression of cell specific markers for 28 days (4 weeks) in both FN- and COL-coated PU scaffolds for all co- and tri-cultures under study (Figures 3, 4). Different cell types showed growth, which was dependent on the type of ECM proteins used to coat the scaffolds. More specifically, the presence of FN enhanced the growth of cancer cells (PANC-1) within the multicellular systems (co-culture and tri-culture), while COL assisted in a more even distribution and higher number of stellate (PS-1) and endothelial (HMEC) cells (Figure 4). It is worth noting that previous published research, wherein such multicellular models consisting of cancer, endothelial, and stellate/fibroblast cells were attempted, a depletion of the supporting cells (endothelial and fibroblast/stellate) was observed at a very early stage of culture (day 4) (Di Maggio et al., 2016; Lazzari et al., 2018). In contrast, our polymer scaffold-based model was successful in maintaining the complex multicellular model of PDAC for 28 days (4 weeks).

### Novel Hybrid, PU Scaffold-Based Multicellular PDAC Model

Based on our observations above (section “Single Homogeneous Scaffold-Based Multicellular Model of PDAC”), it was evident that different cell types within the tumor niche prefer different ECM proteins for high growth and survival. Hence, to account for this, we designed a novel hybrid, multi-compartmental multicellular model consisting of (i) an external/outer collagen-coated cuboid compartment for growth of the stromal cells, i.e., stellate and endothelial cells, and (ii) an internal/inner FN-coated cylindrical compartment for growth of the pancreatic cancer cells (Figure 1). We observed cell growth and proliferation (Figure 9), presence of cell-specific markers (Figure 10), the production of COL (Figure 11), as well as cell migration (Figure 12) within our novel hybrid model over a period of 35 days (5 weeks). Previous studies focusing on multicellular, *in vitro* models of pancreatic cancers have all been spheroids/cell aggregate based and were maintained in culture for a relatively short time period, i.e., between 24 h and 7 days (Froeling et al., 2009; Di Maggio et al., 2016; Ware et al., 2016; Lazzari et al., 2018). To the best of our knowledge, we report here for the first time, a long-term (35-day) PU scaffold-based, hybrid, zonal, multicellular (cancer, stellate, and endothelial cells) model of the PDAC tumor niche.

#### Characterization of Separate Inner (PANC-1) and Outer Compartment (HMEC, PS-1) Compartments of the Hybrid Scaffold

Prior to the development of the hybrid zonal scaffold, we studied independently the two scaffold compartments of the hybrid scaffold to monitor long term the evolution of the three different cell types (Figure 1).

##### Inner fibronectin-coated cylinder scaffold compartment (PANC-1 cells)

We have previously demonstrated that PANC-1 cancer cells prefer FN-coated PU scaffolds for long-term cell proliferation and for mimicking various *in vivo* characteristics like COL production, realistic hypoxic gradients, and treatment resistance (Totti et al., 2018; Gupta et al., 2019). Hence, we cultured PANC-1 cancer cells on FN-coated cylindrical PU scaffolds for 28 days [see also sections “Scaffold-Based Zonal 3D Cell Culture” and “Fibronectin-Coated PU Inner Cylinder Compartment of the Hybrid Scaffold (Containing PANC-1 Cells)”].

As shown in Figure 5, PANC-1 cancer cells were able to proliferate within the FN-coated cylinder scaffold compartment for the entire duration of the experiment. We also monitored the secretion of COL as it is an important feature of the PDAC TME *in vivo* (Apte et al., 2004; Armstrong et al., 2004; Shintani et al., 2006; Shields et al., 2011). We observed COL production by the PANC-1 cancer cells as early as 14 days post-cell seeding, which increased throughout the culture period (Figure 5). These observations are in agreement to our previously published monocellular model of PDAC on FN-coated PU cubic scaffolds (Totti et al., 2018). Furthermore, with respect to the upregulation of cell-specific markers, PANC-1 cells contained a heterogeneous mixture of cells positive for both pan-Cytokeratin and CD-24 throughout the entire culture period (Figure 5), indicating that the PANC-1 cells were able to maintain their neoplastic characteristics long term.

##### Outer collagen-coated cuboid compartment (PS-1, HMEC cells)

As observed in the mono-culture study (Figure 2A), HMEC endothelial cells preferred COL-coated scaffolds over uncoated or FN-coated ones. This is in agreement to previously published literature, wherein endothelial cells’ preference for COL matrix over other materials, like alginate and fibrin, has been reported (Rioja et al., 2016; Nguyen et al., 2017). PS-1 stellate cells showed a preference for coated scaffolds over those uncoated (Figure 2B) but did not show any specific preference for either COL or FN. Froeling et al. (2009) have reported a similar observation wherein PS-1 cells grew similarly in presence of collagen, FN, and Matrigel. Therefore, COL was selected to coat the external stromal compartment of the hybrid scaffolds. As described in Section “Scaffold-Based Zonal 3D Cell Culture,” three different rations of stellate and endothelial cells were studied. Ki-67 positive proliferative cells were present in all three cell ratios under study (1:1, 2:2, and 2:9; HMEC:PS-1) throughout the entire culture period (35 days). However, we observed a decrease in the total cell number toward the end of the culture period, i.e., from day 28 days onward (Figure 6). As observed in Figure 7, for the 1:1 and 2:2 cell ratios (i.e., the conditions with equal number of HMEC and PS-1 cells), both HMEC and PS-1 cell were present within the PU scaffolds. PS-1 stellate cells had aligned fibril cellular morphology, which supports their active state (Bachem et al., 1998; Masamune et al., 2003). CD-31 positive HMEC cells were visible within the PS-1 fibrous stroma. These cellular markers and close interactions between PS-1 and HMEC cells were clearly observed until day 21 of culture. However, on day 28 and beyond, we observed changes in the cellular morphology of the PS-1 cells, i.e., a loss of their fibril-like structure (Figure 7). We also observed a decrease in cell number, loss of cell-specific markers, and a separation of the two cell types, which could be attributed to the natural aging of the cells. We have previously observed a similar cellular aging within our mono-culture model (PANC-1 cells only), wherein a decrease in cell number was seen after 28 days of culture (Gupta et al., 2019). However, it is difficult to compare our observations with existing literature as, to the best of our knowledge, there are no similar long-term (35-day) studies. For the 2:9 cell ratio, wherein an abundance of PS-1 stellate cells were present, the fibrous cellular morphology of the stellate cells was observed as early as day 7 (Figure 7). Due to the abundance of stellate cells, the growth of HMEC endothelial cells was reduced within this system, and a relatively low number CD-31 positive cells were observed (Figure 7). Overall, we did not observe any sprouting and vessel formation within our co-culture, which may suggest a need for more specialized media containing growth factors promoting angiogenesis like VEGF, or those found in Matrigel, to promote structured angiogenesis (Gerhardt et al., 2003; Son et al., 2006; Eichmann and Simons, 2012; Siemerink et al., 2012; Yin et al., 2018). It should be highlighted that although we observed some degree of cellular aging from day 28 (4 weeks) onward, both cell types (HMEC and PS-1) were present within our COL-coated PU cuboid scaffold compartment for 35 days (Figure 7), which is significantly longer than currently reported co-cultures of stellate cells and endothelial cells (Di Maggio et al., 2016). More specifically, Di Maggio et al. (2016) developed a hydrogel-based system consisting of Matrigel and COL, wherein co-culture of PS-1 stellate cells and HUVECs, as well as the effects of PS-1 stellate cells on HUVECs, were assessed for 96 h. In that system, the presence of stellate cells along with collagen and Matrigel assisted in endothelial cell sprouting and the formation of a luminal structure. Generally, activated pancreatic stellate cells have been well-established to be the key element behind the ECM-rich (primarily COL), fibrotic/desmoplastic TME of pancreatic cancer (Apte et al., 2012; Suklabaidya et al., 2018). To assess the PS-1 stellate cells’ capability of mimicking this desmoplastic feature in our system, human-specific COL immunostaining was carried out. High amounts of COL were observed within our outer cuboid scaffold compartment for all three cell ratios under study (Figure 8). Furthermore, COL showed aligned structures (Figure 8), which are known to support/promote metastasis of pancreatic cancer cells (Drifka et al., 2016). Thus, we demonstrated successfully the development and long-term (35-day) maintenance of endothelial and stellate cells scaffold-assisted co-culture, which can act as a “supporting” compartment for our novel hybrid tri-culture model of PDAC. To the best of our knowledge, this is the longest reported co-culture of stellate cells and endothelial cells in a 3D *in vitro* model.

#### Characterization of Hybrid, Scaffold-Assisted Multicellular Model of PDAC

Following the assessments of the independent inner and outer scaffold compartments, the complete hybrid zonal *in vitro* model of PDAC was assembled and studied. Very few studies are available for multicellular *in vitro* models of PDAC involving cancer cells, endothelial cells, and stellate/fibroblast cells to mimic the fibrosis, and all of these studies were carried out for a relatively short period of time (24 h to 7 days). For example, Beckermann et al. (2008) cultured a multicellular model of PDAC involving MIA PaCa-2 pancreatic cancer cells, primary fibroblasts, and HUVECs in a spheroid system for 24 h. Similarly, Lazzari et al. (2018) developed a multicellular spheroid-based model of PANC-1 cancer cells, MRC-5 fibroblasts, and HUVECs and assessed the effects of chemotherapeutic agents (gemcitabine and doxorubicin) within it. The model was viable for 4 days, beyond which loss of HUVECs and MRC-5 fibroblasts was observed. Di Maggio et al. (2016) developed a hydrogel-based tri-culture of PDAC with cancer cells (Capan-1, AsPC-1, and COLO-357), HUVECs, and stellate cells (PS-1). The system was cultured for 7 days. A significant decrease in the number of endothelial cells (HUVECs) in the developed hydrogel-based tri-culture system was observed after 72 h. In contrast to the currently reported spheroid-based studies, our hybrid, PU highly porous scaffold-based, zonal model of PDAC was able to support all three cell types for a total of 35 days (5 weeks) making it the longest reported *in vitro* model of PDAC. Further studies to elucidate the reasons behind the progressive loss of the supporting cells (endothelial and stellate cells) in 3D models would be informative.

As previously described, our novel hybrid scaffold-based multicellular model was characterized via immunostaining and CLSM imaging to assess cell growth and proliferation, ECM protein secretion and maintenance of cellular morphology and phenotypic characteristics (Figures 9–11). We have successfully demonstrated that our hybrid scaffold could maintain proliferating cells (Figure 9) expressing cell-specific markers (Figure 10) throughout the entire culture period (35 days). Furthermore, our model showed extensive COL secretion by the stellate cells and even the cancer cells to some extent, indicating its ability to mimic *in vitro* the PDAC desmoplastic nature (Figure 11). This fibrotic desmoplastic nature of PDAC is a key reason behind the resistance of pancreatic cancer to currently available therapeutic methods; therefore, recapitulating it *in vitro* is key for more accurate treatment screening trials (Chand et al., 2016; Bynigeri et al., 2017; Ansari et al., 2018). We also observed cellular migration across the two zones by all three cell types, highlighting that the cells are able to overcome the physical barrier of being in two separate scaffolds zones (Figure 12). Cellular migration by the cancer cells and the stromal cells, along with cross-talk between them has been linked with PDAC metastasis (Keleg et al., 2003; Xu et al., 2010; Tuveson and Neoptolemos, 2012; Zhan et al., 2017). Hence, this characteristic of our model can be exploited to study the metastatic properties of PDAC. In terms of total cell numbers in our hybrid scaffolds, as expected, differences were observed for different seeding ratios. Nonetheless, the different seeding ratios of the three cell types all showed similar characteristics in terms of cell proliferation (Figure 9), expression of phenotypic markers (Figure 10), and COL production (Figure 11). The choice of seeding density for future work would depend on the specific aim of the work. For example, if the aim would be to study the effect of desmoplasia, then high number of stellate cells (1:2:9 ratio) would be an ideal choice; however, if the aim would be to study more in depth the interactions between the different cell types, conditions with equal number of stellate and endothelial cells would be more appropriate, promoting the presence of higher amounts of endothelial cells. Furthermore, the availability of PDAC models with different ratios of the cells involved is important to account for tumor variability among patients and even intra-tumoral variability for the same patient, since fibrotic intensity as well as vascularization levels differ between patients (Junttila and De Sauvage, 2013; Koay et al., 2016; Verbeke, 2016). Coupled with the feasibility of maintaining a long-term robust culture, our hybrid model’s ability to mimic desmoplasia and to account for tumor/patient variability, highlights the possibility of using it to (i) study the mechanisms behind PDAC’s therapeutic resistance, (ii) assess the effects of therapeutic methods, both traditional (chemo and radiotherapy) (Adcock et al., 2015; Kuen et al., 2017; Al-Ramadan et al., 2018; Gupta et al., 2019) and novel (proton therapy) (Hong et al., 2011, 2014; Terashima et al., 2012), (iii) conduct fractionated radiation screening (Schellenberg et al., 2008; Mahadevan et al., 2010; Loehrer et al., 2011), and (iv) promote personalized treatment screening.

## Conclusion

Overall in this study, we have developed and characterized a novel PU scaffold-assisted multicellular hybrid *in vitro* model of PDAC, with specific ECM protein-coated zones for the tumor compartment and the stromal compartment. More specifically, we have developed, characterized, and maintained for a month a novel tri-culture of pancreatic cancer (PANC-1), endothelial (HMEC), and stellate (PS-1) cells. The inner compartment of the scaffold was FN-coated and contained cancer cells, which were surrounded by an external collagen-coated scaffold compartment consisting of stellate and endothelial cells. Overall, such configuration enabled a more accurate recapitulation of the zonal distribution of different cell types of the pancreatic TME. The developed hybrid zonal model was able to (i) support long-term growth and proliferation of cancer (PANC-1), endothelial (HMEC), and stellate (PS-1) cells for up to 35 days (5 weeks), (ii) allow the maintenance of cell specific morphology and phenotypic markers, (iii) form dense desmoplastic region through abundant sections of COL protein, and (iv) demonstrate cellular migration between the different zones. With the capability of mimicking several key characteristics of the PDAC tumor (desmoplasia, cellular migration), the model shows great potential for future use in a range of applications from basic cancer studies to personalized healthcare. Future work on this model will focus on (i) further validation of the model’s robustness with patient samples, (ii) assessment of the model’s capability to mimic the PDAC’s treatment resistance, and (iii) incorporation of immune cells with the help of perfusion bioreactor.

## Data Availability Statement

The datasets generated for this study are available on request to the corresponding author.

## Author Contributions

PG contributed to conception and design of experiments, conduction of experiments, data collection, data analysis and interpretation, and manuscript writing. PP-M contributed to data interpretation and manuscript reviewing. HK contributed to provision of PS-1 stellate cells and manuscript reviewing. AN contributed to data interpretation and manuscript reviewing. GS contributed to data interpretation and manuscript reviewing. EV contributed to conception of scientific work, data interpretation, manuscript writing and reviewing, and financial support of work.

## Conflict of Interest

The authors declare that the research was conducted in the absence of any commercial or financial relationships that could be construed as a potential conflict of interest.

## Supplementary Materials

FIGURE S1 Representative image of IF staining of outer cuboid with green (PS-1) and red (HMEC) separate channels. Scale bar = 100 μm.
