# Supplementary figures and images for: A Novel Scaffold-Based Hybrid Multicellular Model for Pancreatic Ductal Adenocarcinoma—Toward a Better Mimicry of the in vivo Tumor Microenvironment
Source: Front Bioeng Biotechnol. 2020 Apr 24;8:290. doi: 10.3389/fbioe.2020.00290 (PMC7193232; doi:10.3389/fbioe.2020.00290)

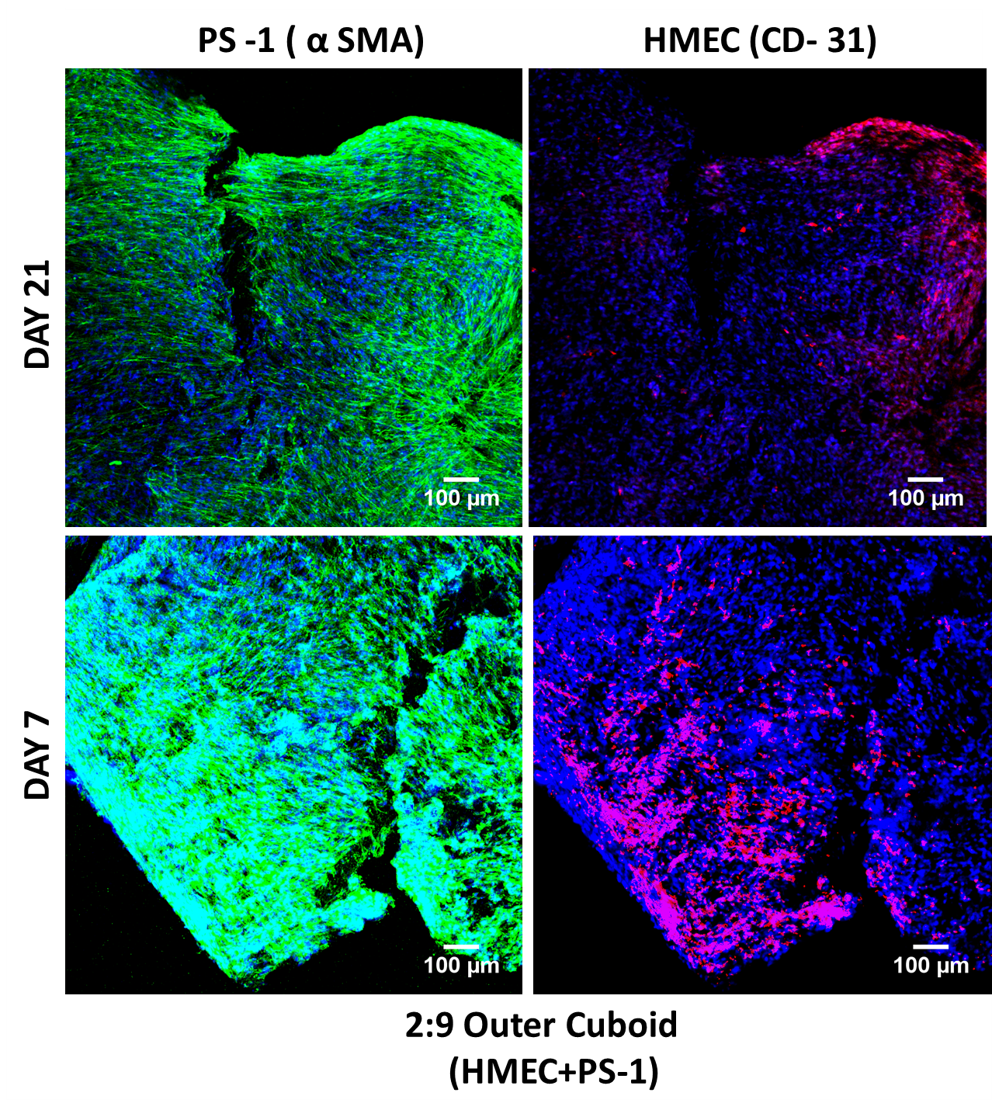

Supplement: FIGURE S1 — Representative image of IF staining of outer cuboid with green (PS-1) and red (HMEC) separate channels. Scale bar = 100 μm. [file Image_1.tif]
